# A Novel Organic Dopant for Spiro-OMeTAD in High-Efficiency and Stable Perovskite Solar Cells

**DOI:** 10.3389/fchem.2022.928712

**Published:** 2022-07-25

**Authors:** Ying Guo

**Affiliations:** School of Materials and Energy, University of Electronic Science and Technology of China, Chengdu, China

**Keywords:** perovskite solar cells, hole-transporting materials, device performance, organic dopants, hydrophobic, operational stability

## Abstract

Perovskite solar cells (PSCs) have achieved excellent power conversion efficiencies (PCEs); however, there still exist some major challenges on device stability due to hydrophilic bis(trifluoromethane)sulfonimide lithium (Li-TFSI), which is commonly introduced as a p-dopant to increase the hole mobility and conductivity of 2,2′,7,7′-tetrakis-(N,N-di-4-methoxyphenylamino)-9,9′-spirobifluorene (spiro-OMeTAD) hole-transporting materials (HTMs). Ion migration, corrosiveness, and hygroscopicity induced by the additive Li-TFSI are detrimental to the device stability, which significantly hinders further commercialization of PSCs. Herein, a hydrophobic organic ionic compound, trityltetra(pentafluorophenyl)borate (TPP), is explored as a novel efficient and stable alternative p-dopant, avoiding the long-term aging process to improve the conductivity of spiro-OMeTAD. As a result, the champion efficiency of TPP-based devices delivers performance up to 23.03%, which is higher than that of the Li-TFSI–based devices (22.39%). In addition, the TPP-based devices also exhibit higher average PCE values. The excellent performance with TPP may be associated with the higher work function of doped spiro-OMeTAD and a better alignment of energy levels with the valence band of perovskite, which substantially accelerate interfacial carrier transportation and minimize the open-circuit voltage (*V*_oc_) loss of PSCs. More importantly, the un-encapsulated TPP-doped devices also display much superior operational stability under maximum power point (MPP) tracking with continuous light illumination in an ambient humid environment, which maintained 96–97% of the initial PCE over 1,100 h outputting. Thus, this work will open up new possibilities for hydrophilic Li-TFSI dopant replacements.

## Introduction

Perovskite solar cells (PSCs) have become the most potential devices because of the outstanding features of organic–inorganic hybrid halide perovskite materials, such as high charge carrier mobility (Motta et al., 2015), extended charge diffusion length (Yang Z. et al., 2019), and excellent light absorption (Chen Z. et al. 2017). Over the past decade, the power conversion efficiency (PCE) of PSCs has risen from 3.8% (Kojima et al., 2009) to the currently certified 25.7% (NREL). Despite the great progress in photovoltaic performance, there are indeed various obstacles in long-term stability for this type of solar cells which limit their large-scale commercialization, such as the notorious moisture sensitivity of perovskite materials (Qiu et al., 2021). Tremendous efforts have been dedicated to alleviating these stability-limiting factors of perovskite materials while maintaining high efficiencies, such as additive engineering (Zhang and Zhu., 2020), defect passivation (Jiang Q. et al., 2019), and component optimization (Rong et al., 2015).

In the most prevalent architecture (n-i-p) of PSCs, hole-transporting layers (HTLs) play an important role in the promotion of charge extraction, transportation as well as photovoltaic performance. Despite the fact that various alternatives for hole-transport materials (HTMs) have been developed, the state-of-the-art 2,20,7,70-tetrakis-(N,N-di-4-methoxyphenylamino)-9,90-spirobifluorene (spiro-OMeTAD) has been demonstrated to be one of the most adequate small molecules as HTM due to its amorphous nature, high solubility, and appropriate highest-occupied molecular orbital (HOMO) energy level (Rombach et al., 2021). Nevertheless, the raw spiro-OMeTAD film suffers from serious drawbacks such as inferior hole mobility and rough surface with pinholes and cracks, which hinder effective charge transport and induce oxygen and moisture to permeate into the perovskite layer, thus causing severe device deterioration. Besides, the energy level offset between the HOMO of the spiro-OMeTAD and the valence band maximum (VBM) of the perovskite still exists. As a result, the photogenerated holes will accumulate at the perovskites/HTL interface, increasing undesirable charge recombination as well as reducing the hole injection efficiency. Therefore, the short-circuit current density (*J*_sc_) and fill factor (FF) may decline accordingly to some extent. Generally, bis(trifluoromethane)sulfonimide lithium (Li-TFSI) and 4-tert-butylpyridine (*t*-BP) are used as additives in spiro-OMeTAD to address these issues. The concentration of oxidized spiro-OMeTAD can be increased remarkably in a humid air environment by introducing a Li-TFSI dopant, bringing the Fermi level of spiro-OMeTAD closer toward its HOMO, thus increasing its conductivity. As for the *t*-BP, it can optimize the morphology and homogeneity of Li-TFSI–doped spiro-OMeTAD HTL. Even though these two prevalent dopants are commonly used in most record-breaking PSCs, their intrinsic physical characteristics have a direct impact on device stability. Considering that the Li-TFSI and *t*-BP cannot oxidize spiro-OMeTAD without oxygen, it is a common practice to age Li-TFSI–doped spiro-OMeTAD in a warm humid environment overnight or for several weeks to obtain higher efficiency, which may lead to poor reproducibility and device instability. In addition, the hygroscopic Li-TFSI absorbs moisture from the atmosphere and aggregates in the spiro-OMeTAD film, deteriorating the interfacial band alignment, interface adhesion, and film morphology. Besides, the Li-TFSI can easily migrate through the octahedral halide cage due to its small radius of Li^+^ cation and TFSI^−^ anion. The migration of ions could act as a random dopant and change the charge equilibrium of the whole device, thus accelerating the degradation of PSCs during aging. Moreover, migrating Li^+^ may be collected at the interface of perovskite/ETL, negatively impacting the efficiency and hysteresis (Li et al., 2017b). Because of its low boiling point, *t*-BP could evaporate easily and form pinholes in the HTLs, and the interfacial *t*-BP chemically decomposes perovskite films by forming a coordinated complex of [PbI_2_-*t*-BP] in the case of long-term storage (Kim et al., 2016). Hence, the addition of Li-TFSI and TBP promotes moisture/oxygen penetration into perovskite layers, which can severely damage perovskite materials, hastening the breakdown of PSCs. Therefore, the exploration of substitute dopants for Li-TFSI and *t*-BP in spiro-OMeTAD is urgent and challenging. Some promising dopant-free HTMs have also been designed to improve device stability, but the efficiency is substantially lower than that of traditional HTMs containing additives or dopants (Miyasaka et al., 2020). Recently, several promising materials like 2,3,5,6-tetrafluoro-7,7,8,8-tetracyanoquinodimethane (F4TCNQ) (Huang et al., 2016), PFPPY (Sathiyan et al., 2020), and tris(pentafluorophenyl)borane (BCF) (Reddy et al., 2019) have been reported as p-type dopants for spiro-OMeTAD. Among these dopants, Co (III) complexes (Chen C. et al. 2017) are also considered to be good candidates to improve both the efficiency and reproducibility of the PSCs when co-doping with Li-TFSI (Schloemer et al., 2019). However, devices with Co-TFSI dopants typically cannot obtain desirable FF and PCE without Li-TFSI co-doping. Additionally, some of these dopants have low solubility in chlorobenzene, giving rise to uneven HTL films, while others have stability issues as well (Murugan et al., 2022).

Herein, a hydrophobic trityltetra(pentafluorophenyl)borate (TPP)–based organic ionic compound was newly developed as an efficient p-dopant by controlling the mole fraction to spiro-OMeTAD. The hydrophobic organic triphenylmethyl radical in TPP possesses much larger radius than Li^+^ ions, which makes it more difficult to migrate through the perovskite crystal lattice to other functional layers and exhibits much higher hydrophobicity than the Li-TFSI dopant. The fluorine (F) elements in TPP could efficiently passivate trap states at the surface or grain boundaries of perovskite *via* the Lewis acid–base reaction (Yang J. et al., 2019). As a result of these obvious benefits, we postulated that the TPP could be a stable and potential dopant for spiro-OMeTAD. Our findings suggest that introducing TPP into HTM solution not only stimulates the spiro-OMeTAD oxidation but also drives down the HOMO of spiro-OMeTAD to improve the band alignment, facilitating the interfacial carrier transportation as ascertained by fluorescence spectrum (PL) and femtosecond transient absorption (fs-TA) measurements. UPS measurements demonstrated that the work function of the spiro-OMeTAD doped with TPP is increased, favorable to augment the open circuit voltage (*V*_oc_) of the device. As a consequence, an impressive maximum PCE of 23.03% (21.8 ± 1.3) with a *J*_sc_ of 24.46 mA/cm^2^, an FF of 0.820, and a *V*_oc_ of 1.149 V are achieved in a 6 mol% TPP-based device (mole ratio relative to spiro-OMeTAD). Furthermore, the TPP-based devices also exhibit superior stability under 1-sun illumination at ambient condition even with a relative high humidity of 40–55%, retaining 96% of the initial PCE after 1,100 h outputting, which is primarily due to the hydrophobic property of organic molecules in TPP and the absence of hydrophilic Li-TFSI.

## Results and Discussion

### Properties of Doped Spiro-OMeTAD

**FIGURE 1:**
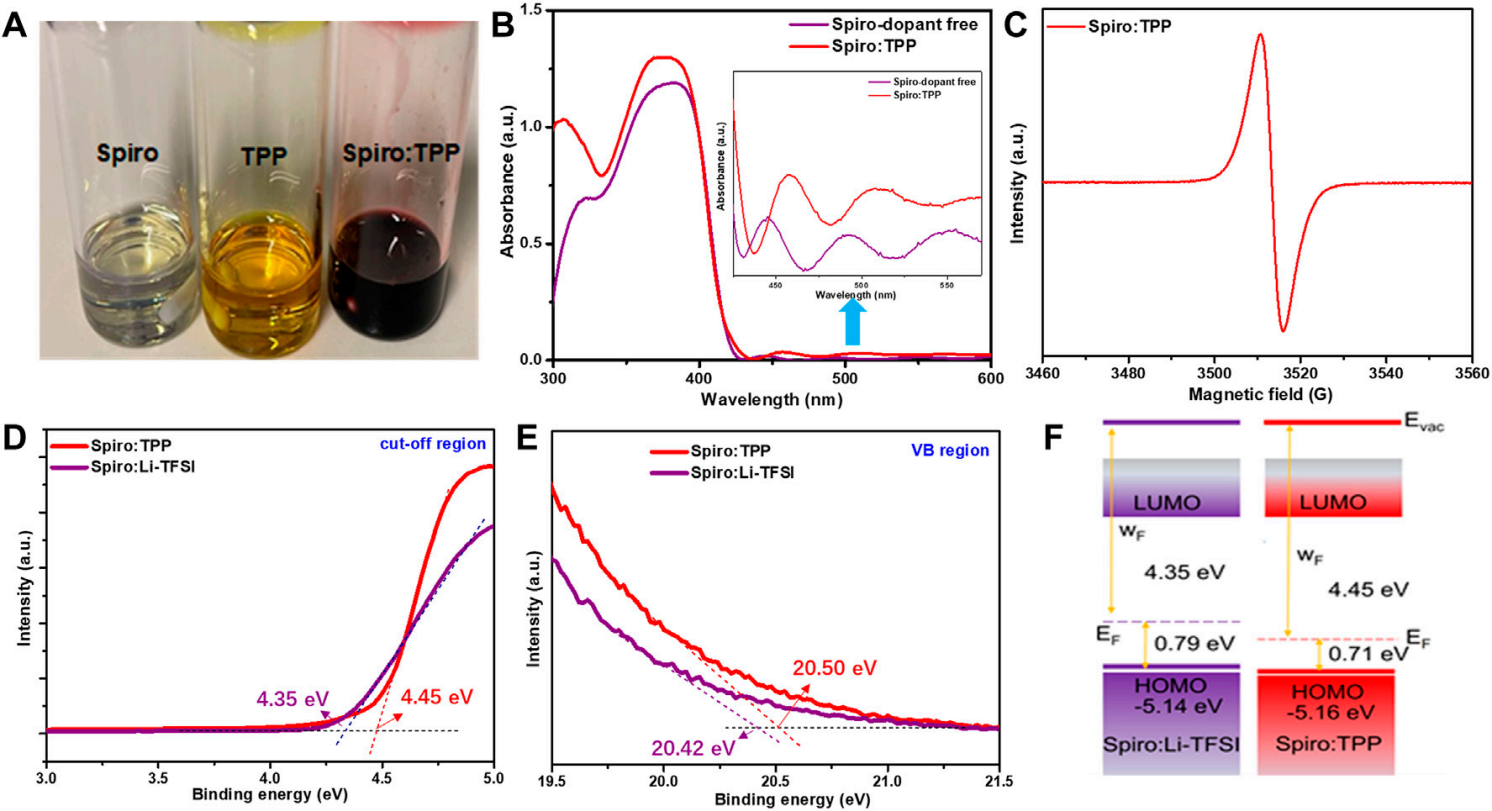
**(A)** Digital photograph of spiro-OMeTAD, TPP, and Spiro:TPP (6 mol%) in the solution of chlorobenzene; **(B)** UV-vis results of spiro-OMeTAD films with or without a TPP dopant; **(C)** ESR measurement of Spiro:TPP; **(D)** UPS of Spiro:TPP and Spiro:Li-TFSI in the electron cutoff region and **(E)** the valence band region; **(F)** energy-level diagram of Spiro:Li-TFSI and Spiro:TPP.

As shown in Figure 1A, it is found that the TPP can be well dissolved in chlorobenzene solution. This organic ionic compound solution appears mustard yellow, while the dopant-free spiro-OMeTAD solution is primrose yellow. Additionally, spiro-OMeTAD solutions doped with the same percentage of Li-TFSI, TPP, Co-TFSI, and Li-TFSI + Co-TFSI are listed in Supplementary Figure S1, and the molecular structure of TPP is exhibited in Supplementary Figure S2. When the TPP is added into spiro-OMeTAD solution, the color turns reddish-brown immediately, which suggests that TPP is critical in triggering the oxidation of spiro-OMeTAD. To verify this hypothesis, UV-VIS-NIR spectrometry (UV-vis) measurements for spiro-OMeTAD with and without TPP dopant were performed (Figure 1B). Apparently, both samples show a characteristic maximum peak at around 390 nm, which is ascribed to the neutral spiro-OMeTAD absorption (Pellaroque et al., 2017). The spiro-OMeTAD doped with TPP (denoted as Spiro:TPP) has a higher absorption from 450 to 550 nm than raw spiro-OMeTAD, implying that the oxidized spiro-OMeTAD may be generated with the incorporation of a TPP dopant (Li et al., 2017a). To explore the possible oxidation effect by TPP, the electron spin resonance (ESR) spectroscopy was then used to probe the free radicals in organic molecules and the result is shown in Figure 1C. Here, a strong paramagnetic signal at around 3510 G was detected for the solution of Spiro:TPP, which implies the emergence of a large number of unpaired mono-cation [spiro-OMeTAD]^+^ radicals after TPP doping, while the ESR of dopant-free spiro-OMeTAD is a flat line without any paramagnetic signal, which has been widely proved in previous ESR results (Dong et al., 2019; Ren et al., 2021; Seo et al., 2021). This phenomenon indicates that fluorine-containing TPP may have a strong electron-accepting ability, prompting the electron transfer from spiro-OMeTAD to TPP. In addition, the concentration of [spiro-OMeTAD]^+^ radicals can change the color of spiro-OMeTAD solution, which is well in line with the aforementioned results. It is speculated that Spiro:TPP does not require a long air oxidation process, and the process can be facilitated by the organic dopant TPP, thus reducing the fabrication time for PSCs.

Conductivity (*σ*) is another important electrical property to measure the performance of HTLs, which could be obtained by direct current–voltage (*I*–*V*) measurements with the structure of FTO/spiro-OMeTAD/Ag (Supplementary Figure S3). The *σ* of HTLs can be calculated by the following equation

$$\sigma =\frac{Id}{VA}$$ (1)

where A is the active area of the device (0.096 cm^2^), d is the thickness of the samples (200 nm), *V* is the applied bias, and *I* is the real-time current. The calculated *σ* for Spiro:dopant-free, Spiro:Li-TFSI, and Spiro:TPP are 3.99 × 10^−4^ S/cm, 2.63 × 10^−3^ S/cm, and 2.79 × 10^−3^ S/cm, respectively. This result indicates that Spiro:Li-TFSI and Spiro:TPP possess a comparable electrical conductivity, higher than that of Spiro:dopant-free.

To probe the influence of TPP doping on the electronic properties of spiro-OMeTAD, the ultraviolet photoelectron spectroscopy (UPS) was carried out to clarify the energy level of spiro-OMeTAD incorporated with TPP or Li-TFSI. The valance band (VB) region (*E*_VB_) and the cutoff (*E*_cutoff_) region of UPS are exhibited in Figures 1D,E. HOMO energy levels could be calculated according to the UPS spectra by the following equation: HOMO = 21.21 eV− (*E*_VB_ − *E*_cutoff_). Consequently, the Spiro:TPP possesses a higher work function (*W*_F_) value of 4.45 eV than Spiro:Li-TFSI (4.35 eV), which means a more p-type spiro-OMeTAD with higher hole concentration was obtained by incorporating TPP. The corresponding schematic energy level diagram is depicted in Figure 1F. As we know, the *V*_oc_ of the device is determined by the discrepancy of *E*_F_ between electron transport materials (ETMs) and HTMs in PSCs. (Shao et al., 2016). Therefore, it is presumed that deeper *E*_F_ of Spiro:TPP-based devices will exhibit higher *V*_oc_ in PSCs. Furthermore, when TPP is introduced, the calculated HOMO of spiro-OMeTAD declines from 5.14 to 5.16 eV, approaching the valence band maximum (VBM) of the perovskite layer. This adjustment of energy levels optimizes VB alignment at the perovskite/HTL interface and improves the perovskite-to-HTL hole transportation at the same time. These findings demonstrate the potential of TPP as a dopant for regulating the interfacial energy levels and rapid oxidation of spiro-OMeTAD HTM.

### Charge Carrier Kinetics and Passivation

**FIGURE 2:**
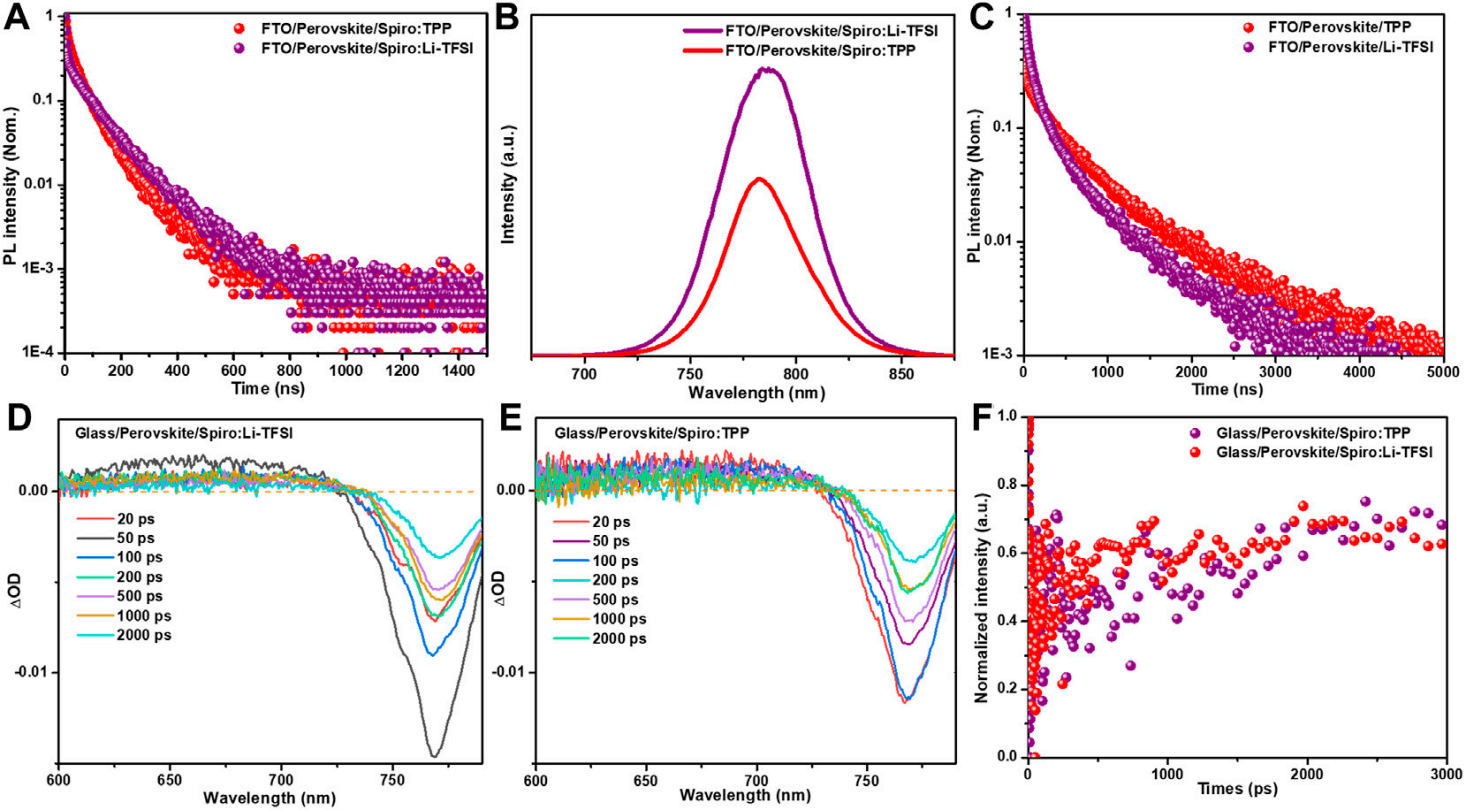
**(A)** TRPL and **(B)** PL for FTO/perovskite/Spiro:TPP and FTO/perovskite/Spiro:Li-TFSI substrates, **(C)** TRPL for FTO/perovskite/TPP and FTO/perovskite/Li-TFSI, **(D)** Fs-TA spectra of glass/perovskite/Spiro:Li-TFSI, **(E)** Fs-TA spectra of glass/perovskite/Spiro:TPP samples with a range time scale of 20–2000 ps, and **(F)** normalized kinetic traces for photo bleaching probed at around 769 nm.

The interface charge transfer at perovskite/HTM is decisive for efficient PSCs manufacturing. To investigate the interface carrier transport kinetics from perovskite to HTM, time-resolved photoluminescence (TRPL) spectroscopy was employed. The FA_0.95_MA_0.05_PbI_2.85_Br_0.15_ double-cation halide perovskite was designated as light absorber in this work, and the perovskite layer was fabricated using a one-step spin-coating method. Figure 2A shows the TRPL spectra based on FTO/perovskite/Spiro:TPP and FTO/perovskite/Spiro:Li-TFSI layers. The decay times were estimated by a *bi*-exponential function (Supplementary Table S1), and the short lifetime *τ*_1_ represents non-radiative recombination from defects, whereas the long lifetime component *τ*_2_ is associated with radiative recombination in bulk perovskite. For FTO/perovskite/Spiro:Li-TFSI film, its average lifetime (*τ*_ave_) of photoluminescence decay is 77.2 ns, with *τ*_1_ = 8.2 ns and *τ*_2_ = 91.7 ns. In case of Spiro:TPP, the lifetimes of both *τ*_1_ and *τ*_2_ decreased to 7.4 and 61.5 ns, respectively, and the corresponding *τ*_ave_ declined to 55.9 ns. The TRPL analysis shows that the Spiro:TPP can promote hole extraction and transportation more effectively. In addition, the steady-state fluorescence (PL) measurements display the same quenching tendency (Figure 2B). The PL intensity of FTO/perovskite/Spiro:TPP was dramatically quenched which further verified the better hole extraction properties of Spiro:TPP HTL (Figure 2B). Besides, the easily observed blue shift of the PL peak from 786 to 783 nm after introducing a TPP dopant indicates that defects on the perovskite surface may also be passivated (Li et al., 2019; Gao et al., 2020; Jiang et al., 2021). To further validate the role of TPP dopants in the passivation of perovskite surfaces, we compared glass/perovskite/Li-TFSI and glass/perovskite/TPP-based TRPL decay in Figure 2C, and fitting results are exhibited in Supplementary Table S1. The results of the TPP-based sample exhibit longer recombination lifetime (*τ*_2_) of 432.1 ns than the Li-TFSI sample (358.3 ns), indicating that the perovskite film has less defects and better quality. Supplementary Figure S4 shows the dark *J–V* measurements of hole-only devices displaying the trap-filled limit voltage (*V*_TFL_) kink point behavior. The architecture of FTO/NiO/perovskite/Spiro:Li-TFSI/Au and FTO/NiO/perovskite/Spiro:TPP/Au were measured to evaluate the trap density in perovskite. The trap density can be calculated using the following equation

$$V_{TFL}=\frac{en_{t}L^{2}}{2\varepsilon \varepsilon_{0}}$$ (2)

where *V*_TFL_ is the onset voltage of trap-filled limit voltage, e is the elementary charge (1.6 × 10^–19^ C), n_t_ is the trap-state density, L is the thickness of perovskite film, *ε* is the permittivity of perovskite, and $\varepsilon_{0}$ is the vacuum permittivity. The obtained trap-state density of perovskite with Spiro:TPP was 6.80 × 10^15^ cm^−3^, which was less than the value of HTL-only device with Spiro:Li-TFSI (7.83 × 10^15^ cm^−3^). This is probably due to the incorporation of TPP, which could passivate the trap states on the surface of perovskite. As spiro-OMeTAD is unable to passivate the defects, the passivation effect on the perovskite surface may be due to the F elements contained in TPP, which has been widely demonstrated in earlier research (Wang et al., 2020).

The structures of glass/perovskite/Spiro:Li-TFSI and glass/perovskite/Spiro:TPP were also applied using femtosecond transient absorption (fs-TA) spectra to gain a better understanding of hole separation and recombination at the interface of perovskite/spiro-OMeTAD. Figures 2D,E exhibit the key ΔOD with decay time ranging from 20 to 2000 ps. All the fs-TA spectra exhibit a positive excited-state absorption (ESA) and a negative ground-state bleaching (GSB) band. The ESA band before 700 nm represents the absorption of perovskite, and the GSB at about 769 nm for both samples is attributed to the fluorescent band-to-band emission of perovskite film. By comparing the 769-nm peak in the GSB band, it is obvious that the perovskite film based on Spiro:TPP shows a faster decay at different delay times than that of the Spiro:Li-TFSI, in accordance with the quenching phenomena in PL measurements. This indicates that the extraction of photogenerated holes could be more efficient from perovskite to HTL. Figure 2F shows the time evolutions of the GSB peaks near 769 nm for Spiro:TPP and Spiro:Li-TFSI to investigate the carrier kinetics of hole extraction. The ultra-high speed decay scale implies a favorable extinction of charge extraction or injection (Tress, 2017), which can be fitted by a double exponential decay model. In this model, the fast decay component in the picosecond range (<1 ns) is attributed to the process of photogenerated hole extraction from the perovskite layer, and the slow decay component in the nanosecond range (>1 ns) is attributed to trap-assisted recombination at the bulk perovskite and interface (Wang et al., 2014). To explore interface carrier transfer process, the fast component of *τ*_1_ was considered. The sample with the TPP dopant exhibits an accelerated decay in GSB peak and a shortened decay time from the original 898.4 ps for Spiro:Li-TFSI to 307.1 ps for Spiro:TPP, signifying that the TPP dopant greatly improves hole extraction from perovskite to spiro-OMeTAD.

### Photovoltaic Performance and Characterization

**FIGURE 3:**
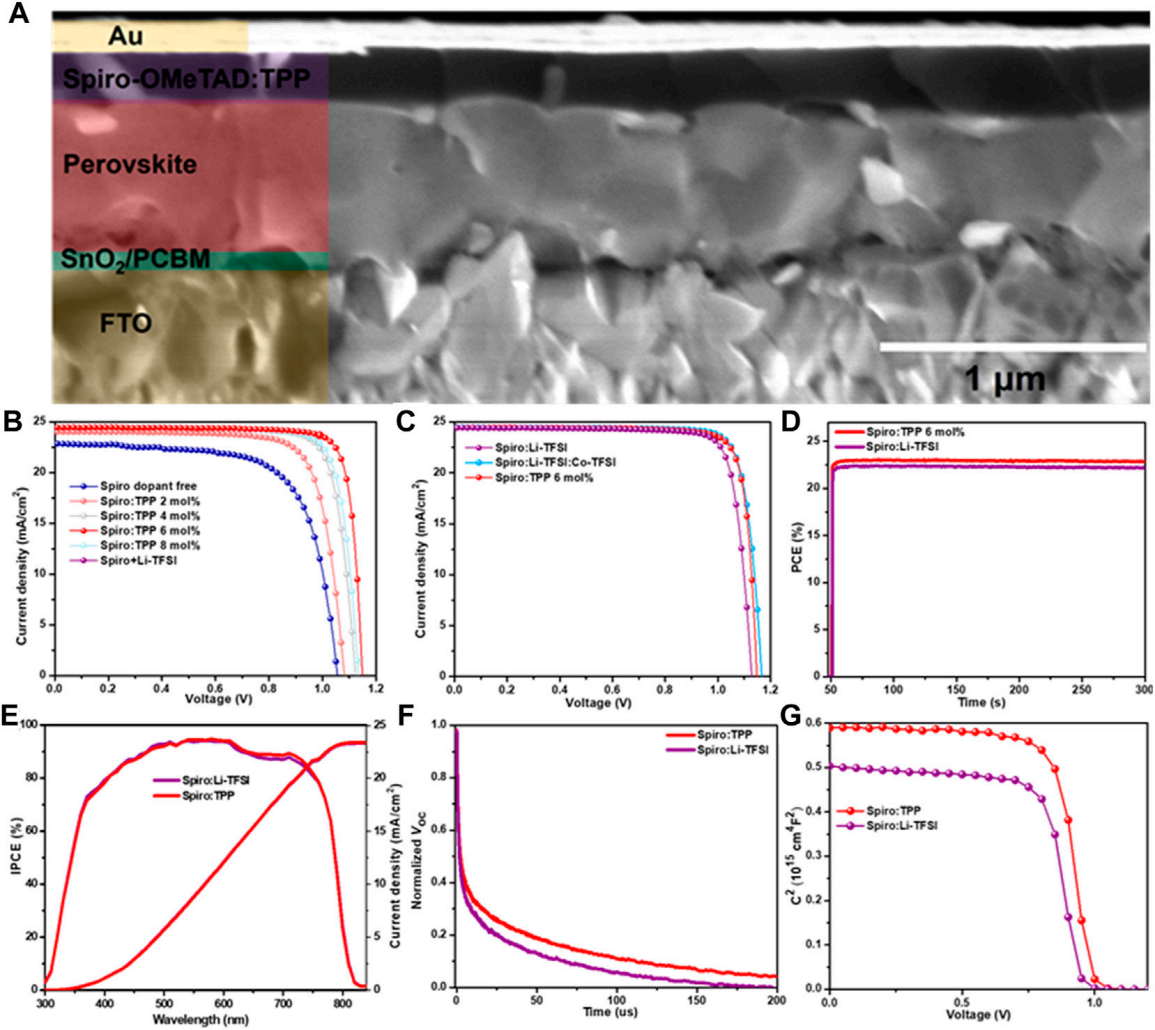
**(A)** Cross-sectional SEM of the whole device with a structure of FTO/SnO_2_/PCBM/perovskite/Spiro:TPP/Au, **(B)** *J–V* curves of champion devices with different concentrations of TPP dopants ranging from 0 mol% to 8 mol%, **(C)** *J–V* curves of champion devices with different dopants, **(D)** steady-state output power at maximum power point (MPP), **(E)** IPCE spectra, **(F)** TPV spectra, and **(G)** Mott*–*Schottky measurements for devices with Spiro:Li-TFSI and Spiro:TPP.

As shown in Figure 3A, the cross-section scanning electron microscopy (SEM) displays the structure of the PSCs based on FTO/SnO_2_/PCBM/FA_0.95_MA_0.05_PbI_2.85_Br_0.15_/Spiro:TPP/Au, and the thicknesses of perovskite layer and spiro-OMeTAD layer are about 600 and 200 nm, respectively. Top-view SEM images of the FA_0.95_MA_0.05_PbI_2.85_Br_0.15_ perovskite, Spiro:Li-TFSI, and Spiro:TPP films are displayed in Supplementary Figure S5. Figure 3B shows the current–voltage (*J*–*V*) curves of champion devices with various doping concentrations (0, 2 mol%, 4 mol%, 6 mol%, and 8 mol%) of TPP under simulated AM 1.5 G solar irradiation (100 mW/cm^2^), and the detailed parameters are summarized in Supplementary Table S2. There is no treatment after the device fabrication. The statistical results from the same batch of ten devices are exhibited in Supplementary Figure S6. As can be seen, the devices with different concentrations of TPP in spiro-OMeTAD show higher average PCE values than devices with pristine spiro-OMeTAD (0 mol%), which suffer from relatively low photovoltaic performance. In the absence of Li-TFSI, with increasing the mole fractions of TPP, all photovoltaic parameters are gradually improved, reaching an optimum performance at 6 mol% and an increased average PCE of ∼40% by contrast to raw spiro-OMeTAD. Obviously, all the photovoltaic parameters are simultaneously enhanced, especially the *V*_oc_, which is probably attributed to higher *W*_F_ and surface passivation effect by using a TPP dopant. Furthermore, the photovoltaic parameters of PSCs with TPP, Li-TFSI, and Li-TFSI:Co-TFSI *bi*-dopant were compared which were measured without overnight oxidation, and the corresponding *J*–*V* plots and the corresponding parameters are shown in Figure 3C and Table 1. The champion PCE of PSCs based on Spiro:TPP devices achieved a PCE of 23.03% (21.8 ± 1.3) with a high FF of 0.820, *V*_oc_ of 1.149 V, and *J*_sc_ of 24.46 mA/cm^2^ at the optimal concentration of 6 mol%, which is comparable to the performance of Li-TFSI and Co-TFSI *bi*-dopant (23.43%) and is much higher than that of Spiro:Li-TFSI–based devices (PCE = 22.39% (21.2 ± 1.2), *J*_sc_ = 24.42 mA/cm^2^, *V*_oc_ = 1.128 V, and FF = 0.813). The statistical results from the same batch of ten devices are also exhibited in Supplementary Figure S6, which verify the good repeatability of the devices. To ensure the accuracy of the *J*–*V* measurements, the steady-state photocurrent densities (Supplementary Figure S7) and stabilized power output (Figure 3D) tested at the maximum power point (MPP) were recorded over a period of 250 s under AM 1.5 G illumination. As a result, both devices show extremely fast photo response and stabilized PCE. The stabilized photocurrent achieved a PCE of 22.97% at a bias voltage of 1.03 V for Spiro:TPP and 22.31% at 1.01 V for Spiro:Li-TFSI-based PSCs, respectively. The integrated current densities obtained from incident photon-to-current efficiency (IPCE) spectra in the range of 300–850 nm are 23.46 and 23.40 mA/cm^2^ for the PSCs based on Spiro:TPP and Spiro:Li-TFSI (Figure 3E). Except the PCE, the hysteresis behavior in *J*–*V* curves was measured in both reverse and forward scans (Supplementary Figure S8), and detailed photovoltaic parameters are provided in Supplementary Table S3. The hysteresis index (HI) can be estimated by the equation of HI = (PCE_RS_-PCE_FS_)/PCE_RS_, where the PCE_RS_ and PCE_FS_ represent the reverse and forward scans, respectively (Liu et al., 2019). As depicted in Supplementary Figure S8, both the PSCs with Spiro:Li-TFSI and Spiro:TPP have low HI values of 0.045 and 0.031, respectively, while the introduction of TPP presents a lower hysteresis effect. Such a small hysteresis may stem from the decreased defect density in the perovskite/HTL interface and the lack of extrinsic Li^+^ ion migration.

**TABLE 1:** Photovoltaic parameters of devices based on different HTL dopants.

Device	*J*_sc_ (mA/cm^2^)	*V*_oc_ (V)	FF	PCE_max_ (%)	Average PCE (%)
Spiro:Li-TFSI	24.42	1.128	0.813	22.39	21.2 ± 1.2
Spiro:Li-TFSI:Co-TFSI	24.48	1.166	0.821	23.43	22.2 ± 1.3
Spiro:TPP 6 mol%	24.46	1.149	0.820	23.03	21.8 ± 1.3

Furthermore, the decay of the transient photovoltage (TPV) was studied, as shown in Figure 3F. When the device is kept in the open-circuit state with no current extraction, all photogenerated charge carriers recombine, and thus the recombination process reflects the device characteristics. Obviously, the charge recombination lifetime of the Spiro:TPP-based device is higher than that of Spiro:Li-TFSI, which agrees well with the enhanced *V*_oc_. These results indicate that the non-radiative pathway of the TPP-doped HTL is reduced and its ability of charge extracting is improved, which is consistent with the reduced non-radiative recombination from the aforementioned PL results.

Mott–Schottky (M–S) plots were also measured to investigate the interfacial properties’ built-in potential (*V*_bi_) which can be calculated using the following equation: 1/C^2^ = 2(*V*_bi_-*V*)/A^2^qεε_0_N, where A represents the effective area of the device and N is the charge density (Almora et al., 2016). The voltage dependence of 1/C^2^ curves is shown in Figure 3G, and the devices demonstrate a larger *V*_bi_ of 1.01 V by TPP doping than the Spiro:Li-TFSI–based device of 0.97 V, further explaining the significantly increased *V*_oc_ exhibited in *J*–*V* measurements.

### Stability

**FIGURE 4:**
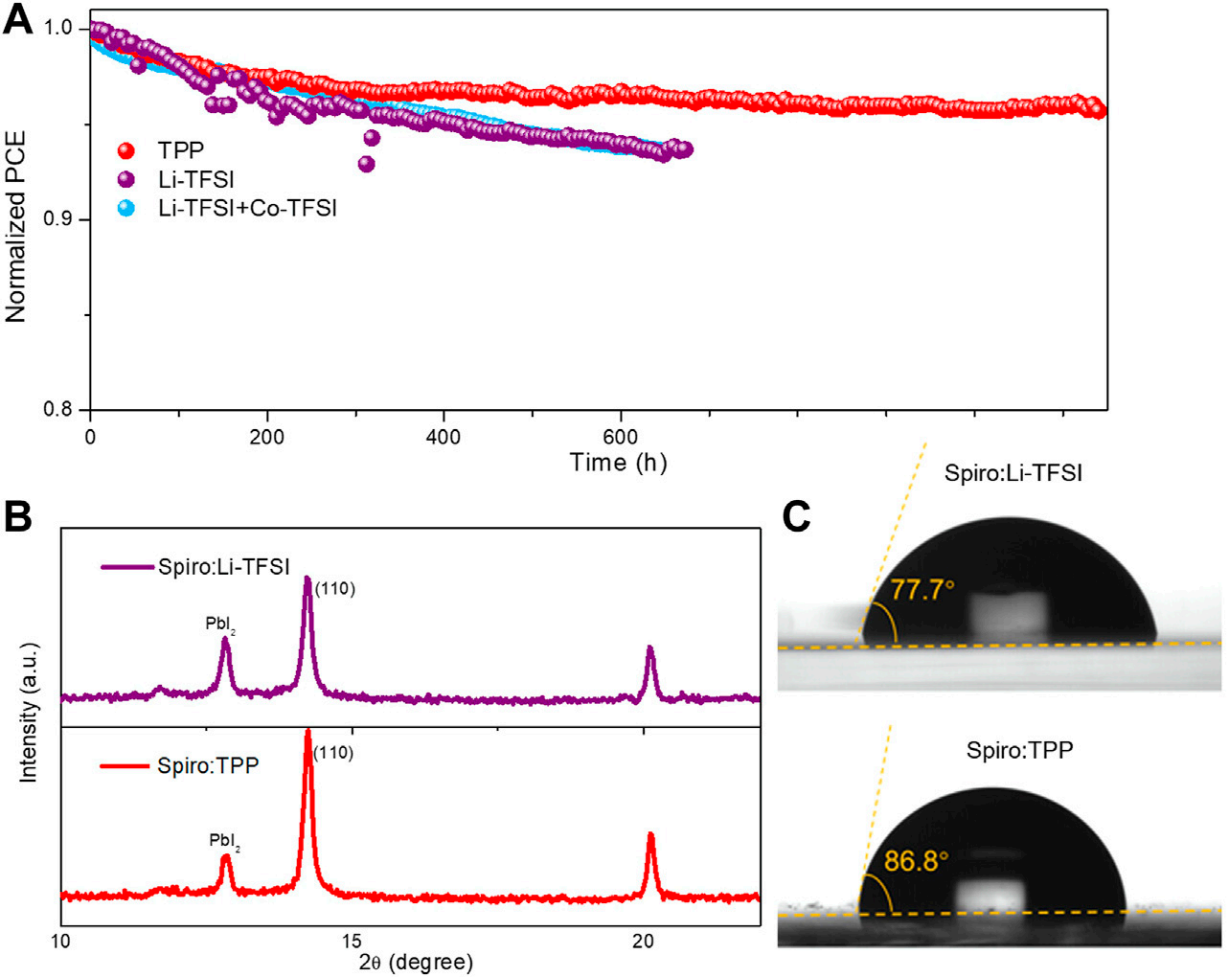
**(A)** The operation stability of devices with Spiro:TPP, Spiro:Li-TFSI, and Spiro:Li-TFSI:Co-TFSI under 1-sun illumination in ambient condition with a relative humidity of 40–55%; **(B)** XRD of devices based on Spiro:Li-TFSI and Spiro:TPP after aging for 700 h; and **(C)** water contact angle for Spiro:Li-TFSI and Spiro:TPP.

The long-term operational stability of the PSCs was explored, as well as the evolution of the normalized PCE with MPP tracking under 1-sun illumination with a relative high humidity of 40–55% at ambient condition. As shown in Figure 4A, the PCE degradation curve for the Spiro:TPP-based device is much slower and retains approximately 96% of its initial efficiency even operating over 1,100 h. In striking contrast, the PCE of devices with Spiro:Li-TFSI drops to 93% of the initial value only operating 600 h under the same aging conditions. In addition, the Li-TFSI and Co-TFSI co-doped devices also exhibit similar stability, exhibiting lower stability than that of TPP which may be attributed to the metal-free of the TPP molecules. To scrutinize the degradation of PSCs, the X-ray diffraction (XRD) was conducted to compare the crystal characteristics of perovskite after aging for 700 h (Figure 4B). After operating in a humid environment for 700 h, both devices showed similar diffraction peak: PbI_2_ (001) at 12.96° and perovskite (110) at 14.34°. Due to the large amounts of water adsorption by the Li-TFSI, the perovskite can partially decompose into PbI_2_. The peak intensity ratio of PbI_2_ (001) to perovskite (110) was utilized as an indicator to evaluate the decomposition degree of the perovskite. Both of the samples exhibit a strong intensity of PbI_2_ which is related to the extra 0.07 M of PbI_2_ in the perovskite recipe. However, the peak intensity ratio declined from 0.523 to 0.201 when TPP was introduced, which implies the TPP will protect the perovskite crystals from moisture. To further verify this speculation, water contact angle measurements were performed. Figure 4C reveals that the film based on Spiro:TPP (86.6°) has a greater and higher contact angle than the films with Li-TFSI doping (77.7°). The higher hydrophobicity of Spiro:TPP with the absence of the hydrophilic Li-TFSI creates a greater shield for water penetration in HTL and hence preserves the inner perovskite from external moisture. Furthermore, thermal stability is another issue for the spiro-OMeTAD–based HTM. Supplementary Figure S9 exhibits the normalized PCE of devices against heating time under 85°C in an N_2_-filled glove box. The device based on Spiro:TPP retained higher initial PCE than that of the device with Spiro:Li-TFSI and Spiro:Li-TFSI:Co-TFSI at the same condition. The enhanced thermal stability with Spiro:TPP may be associated with the elimination of migrating Li^+^ in Li-TFSI (Jiang L.-L. et al., 2019; Bao et al., 2022). These results suggest that TPP is an advanced and promising substitute for a Li-TFSI dopant in improving the stability of PSCs.

## Conclusion

In summary, we have developed a hydrophobic substitute for the hydrophilic Li-TFSI dopant, which delivers high performance up to 23.03% (21.8 ± 1.3) with an increased *V*_oc_ of 1.149 V and a fill factor (FF) of 0.820 without traditional longtime air exposure. The results reveal that TPP has the potential to accelerate the oxidation of spiro-OMeTAD and the excellent performance is attributed to the higher work function and well-matched interface energy level close to the perovskite valence band than the Li-TFSI, favoring the interfacial carrier extraction and transportation. Notably, due to the hydrophobic nature of TPP molecules, the Spiro:TPP-based PSCs also achieved better long-term operational stability in ambient conditions without encapsulation, preserving over 96–97% of its initial PCE after 1,100 h with 40–55% relative humid environment. This work can effectively meet the requirements of excellent device efficiency and stability in photovoltaic applications.

## Data Availability

The original contributions presented in the study are included in the article/Supplementary Material; further inquiries can be directed to the corresponding author.
