# Supplementary material for: A Novel Organic Dopant for Spiro-OMeTAD in High-Efficiency and Stable Perovskite Solar Cells
Source: Front Chem. 2022 Jul 25;10:928712. doi: 10.3389/fchem.2022.928712 (PMC9357902; doi:10.3389/fchem.2022.928712)
Supplement: Supplementary file 1 [file DataSheet1.docx]

**Methods**

**Materials and Solution Preparation**

FAI (99.99%), MABr (99.99%), PbI_2_ (99.99%), PbBr_2_ (99.99%), PCBM, tris(2-(1*H*-pyrazol-1-yl)-4-*tert*-butylpyridine)cobalt(III) (Co-TFSI), bis(trifluoromethane)sulfonimide lithium salt (Li-TFSI), 4-tert-butylpyridine (*t*-BP) and Spiro-OMeTAD (99.95%) were purchased from Xi'an Polymer Light Technology Corp. Trityltetra(pentafluorophenyl)borate (TPP, >98%) was purchased from Aladdin Company. The DMF, DMSO, chlorobenzene (CB) and acetonitrile were obtained from Sigma-Aldrich. The SnO_2_ nanoparticle (15 wt%) was purchased from Alfa. All other solvents and chemicals were obtained from commercial sources and used as received without further purification. The SnO_2_ solution was obtained by diluting the solution in water with a ratio of 1:5. The PCBM was dissolved in CB with a concentration of 10 mg/mL. For the perovskite precursor solution, 1.4 M of FA_0.95_MA_0.05_PbI_2.85_Br_0.15_ made by the FAI, PbI_2_, MABr, PbBr_2_ and MACl (28 mg), as well as with extra 0.07 M of PbI_2_ are dissolved in 1 mL mixed solution of DMF/DMSO (8:1 v/v) then stirred at 60°C for 1 h. The Li-TFSI doped Spiro-OMeTAD (denoted as Spiro:Li-TFSI) solution was prepared by dissolving 72.3 mg Spiro-OMeTAD, 28.8 µL of *t*-BP and 17.5 µL of (Li-TFSI) solution (520 mg Li-TFSI in 1 mL acetonitrile) in 1 mL CB. The Li-TFSI and Co-TFSI co-doped (denoted as Spiro:Li-TFSI:Co-TFSI) solution made by mixed 72.3 mg Spiro-OMeTAD, 28.8 µL of *t*-BP and 17.5 µL of (Li-TFSI) solution (520 mg Li-TFSI in 1 mL acetonitrile), as well as 10 µL Co-TFSI solution (For the Co-TFSI, it made by 375 mg in 1 mL acetonitrile) in 1 mL CB. For TPP doped solutions, the Spiro-OMeTAD (72.3 mg) and TPP were dissolved into 1 mL CB and mixed by desired TPP/Spiro-OMeTAD with a range of mole ratios.

**Devices fabrication**

The pre-cleaned, FTO-coated glass substrates were treated with UV-ozone 30 min before device fabrication, then the SnO_2_ layer was deposited in air condition by spin-coating at 3000 rpm for 30 s, followed by heating at 150 °C for 15 min. Then the substrates were transport to a N_2_-filled glove box. The PCBM layers were deposited on FTO/SnO_2_ substrates via spin-coating PCBM precursor solution at 4000 rpm for 30 s, the layers were then dried at 100 °C for 10 min, the perovskite solution was spin-coated in a two steps program at 1000 and 5000 rpm for 10 and 30 s, respectively. During the second step, 200 μL CB was poured on the spinning substrate 20 s prior to the end of the program, followed by heating at 110 ^o^C for 10 min and then heated by 150 ^o^C for 5 min. The Spiro-OMeTAD solution was deposited on the perovskite layers by spin coating at 3000 rpm for 30 s. Finally, Au electrode was thermally evaporated on the Spiro-OMeTAD-coated film. The device area was 0.09 cm^2^ defined by a shadow mask.

**Characterization**

The ultraviolet photoemission spectroscopy (UPS) measurements were performed by AXIS ULTRA DLD with a HeI monochromator (21.22 eV), The morphologies of the perovskite films were observed by scanning electron microscope (SEM, JEOL JSM-7600F). The femtosecond transient absorption (fs-TA) spectra were performed using a femotosecond regenerative amplified Ti: sapphire laser system (Spectra Physics, Spitfire-Pro) and an automated data acquisition system (ultrafast systems, Helios Fire). The time-resolved PL (TRPL) spectra were measured at room temperature using of time-correlated single photon counting (TCSPC) technique with an excitation wavelength of 474 nm. The steady-state photoluminescence (PL) spectra were performed using a HITACHI (model F-4600) spectrophotometer and the excited wavelength is 460 nm. The *J*-*V* curves of the PSCs were measured by using an electrochemical workstation (CHI 660E, Shanghai Chenhua) under AM 1.5G simulated solar light (100 mW/cm^2^) (CHF-XM-500W, Trusttech Co. Ltd., Beijing, China). The incident light intensity was calibrated with a standard Si solar cell. The incident photon-to-electron conversion efficiency (IPCE) spectra were performed by using a commercial setup (QTest Station 2000 IPCE Measurement System, CROWNTECH, USA). The contact angles were measured by using commercial setup.


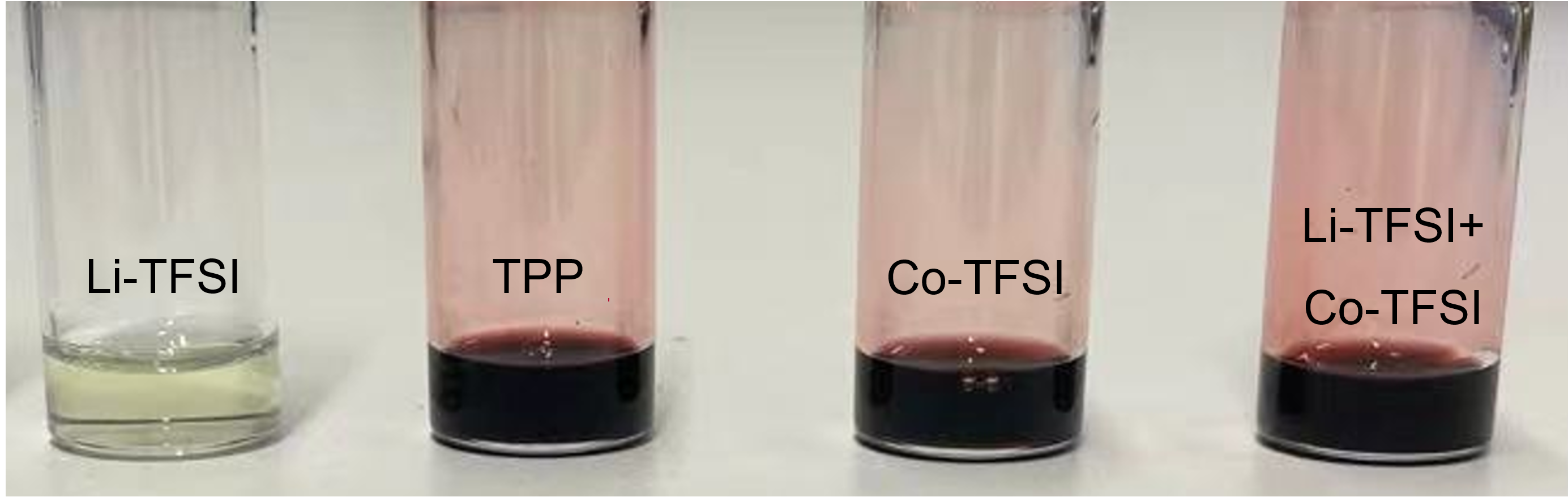


**Figure S1** Photographs of spiro-OMeTAD solutions doped with 6 mol% Li-TFSI, TPP, Co-TFSI and Li-TFSI+Co-TFSI.


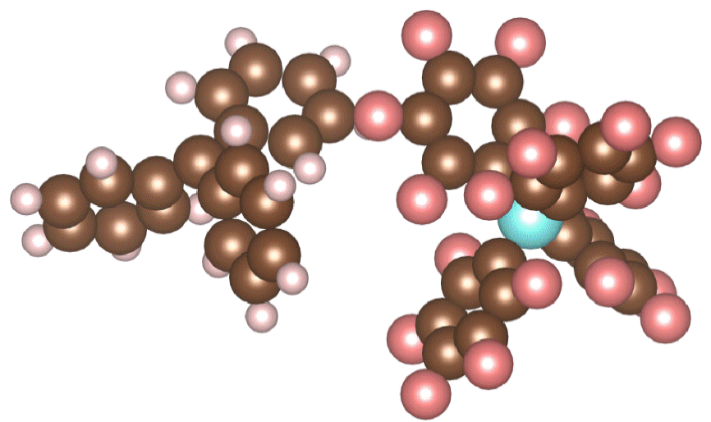


**Figure S2** The structure of TPP dopant, where the brown, light purple, pink and blue colours represents the C, H, F and B atoms, respectively.

**Figure S3** The conductivity measurements for the Spiro-OMeTAD with different dopants.

**Figure S4** Comparison of dark *J-V* curves for HTL-only devices in the architecture of FTO/NiO/perovskite/Spiro:Li-TFSI/Au and FTO/NiO/perovskite/Spiro:TPP/Au.


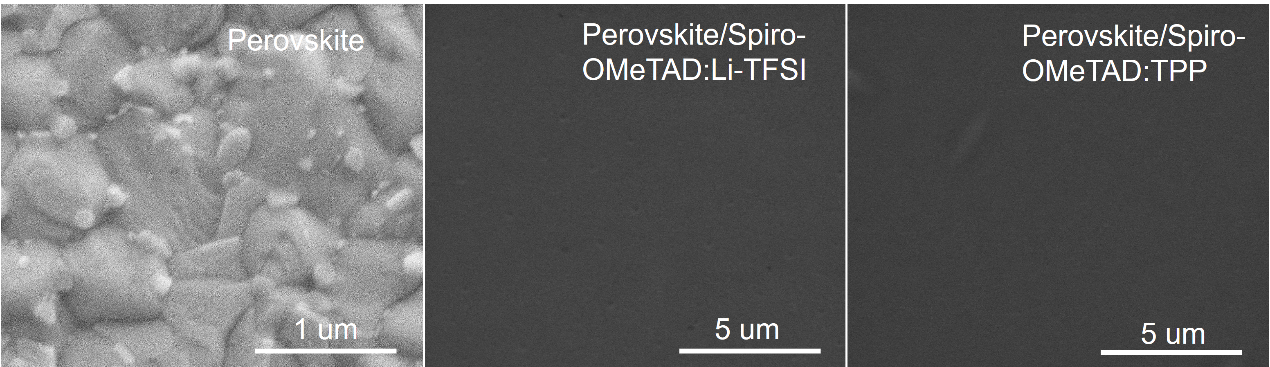


**Figure S5** Top-view SEM images of FA_0.95_MA_0.05_PbI_2.85_Br_0.15_ perovskite, Spiro:Li-TFSI and Spiro:TPP films deposited on perovskite;

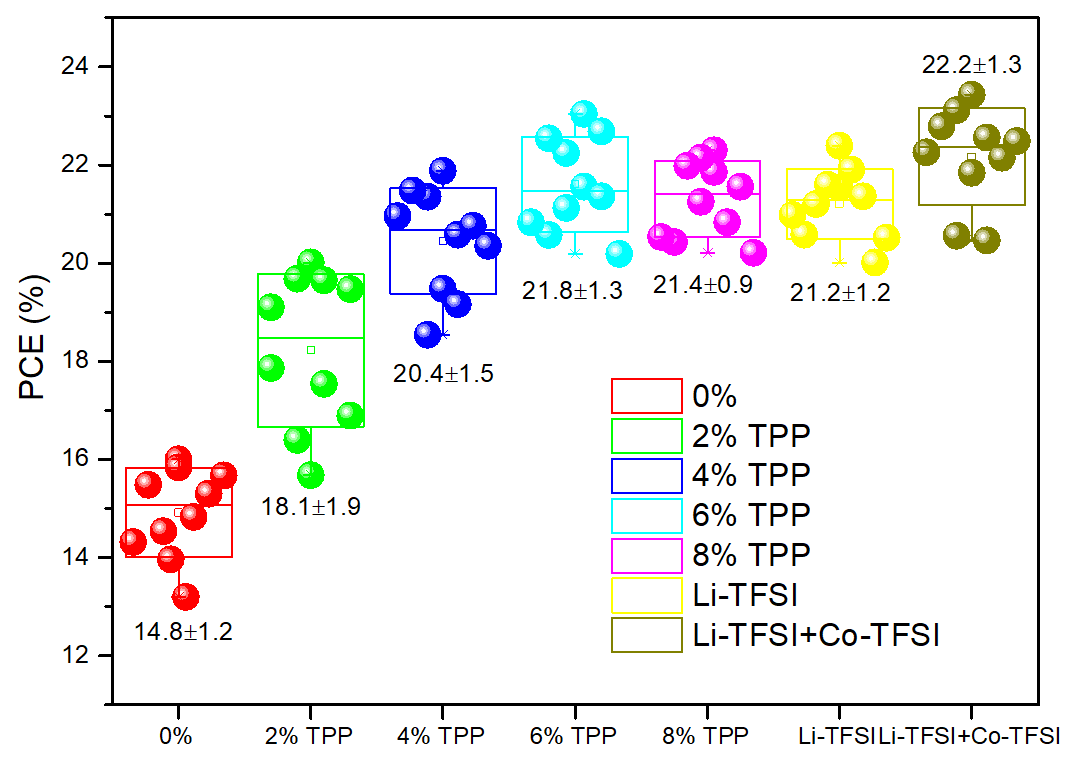


**Figure S6** The statistical *J*_sc_, FF, *V*_oc_ and PCE of devices based on various dopants.


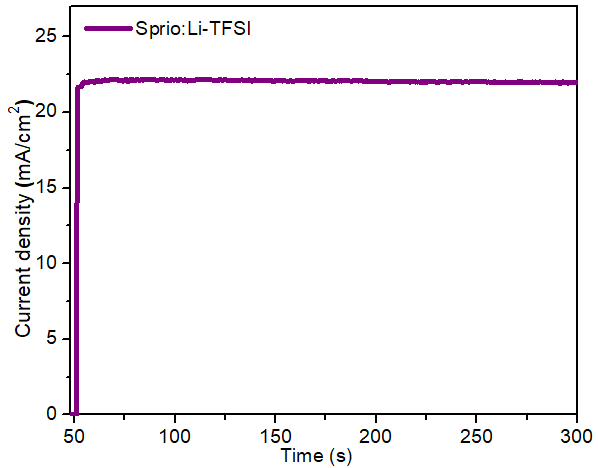

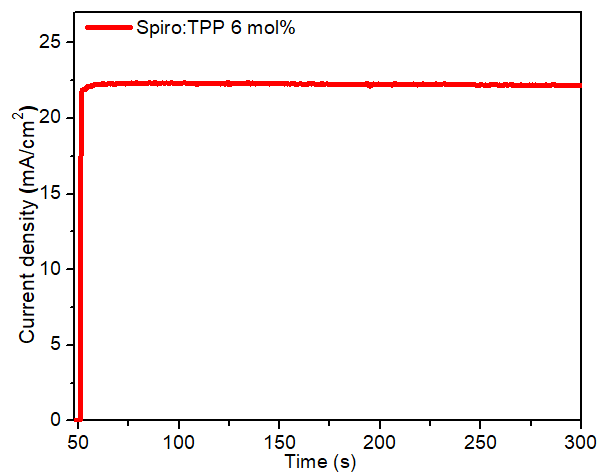


**Figure S7** Stabilized photocurrent density of devices based on Spiro:Li-TFSI and Spiro:TPP measured with the applied voltages of 1.01 V and 1.03 V, respectively.


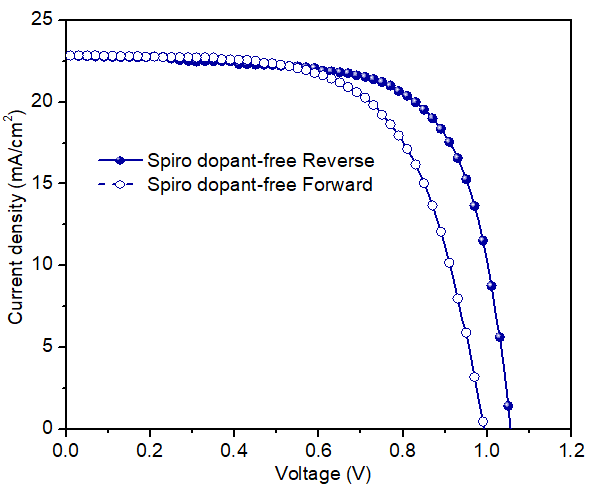

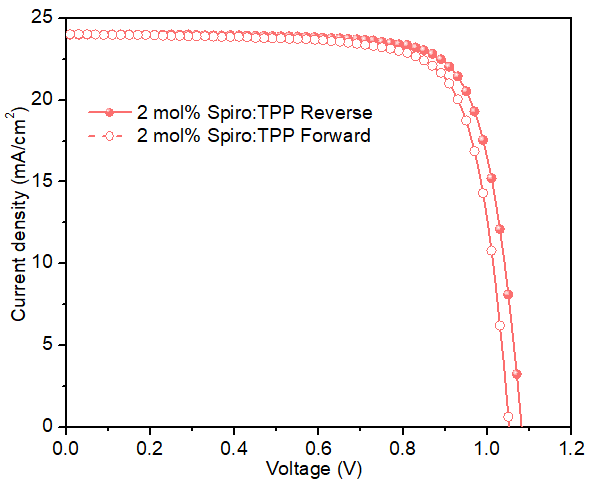


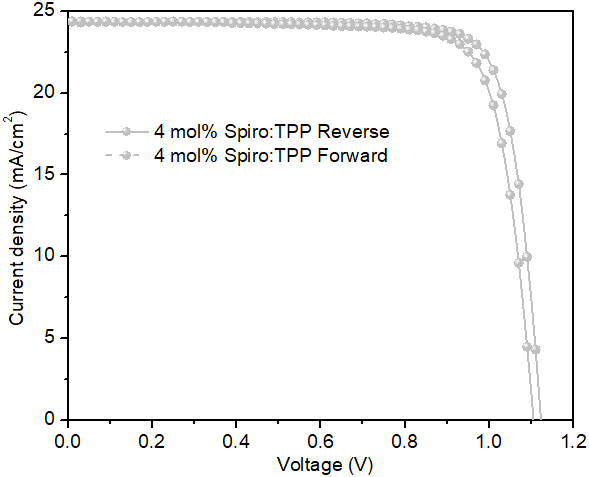

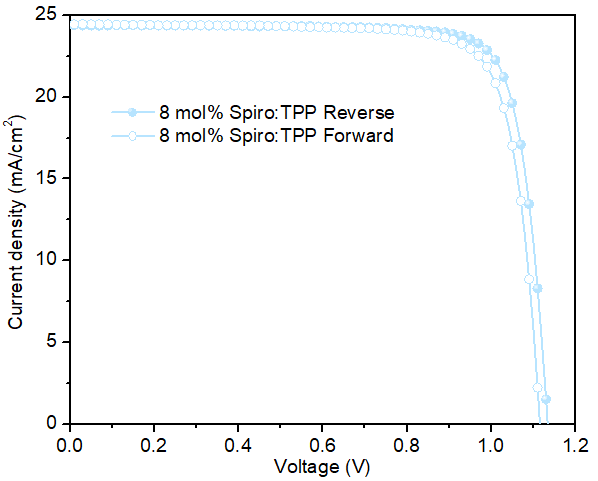


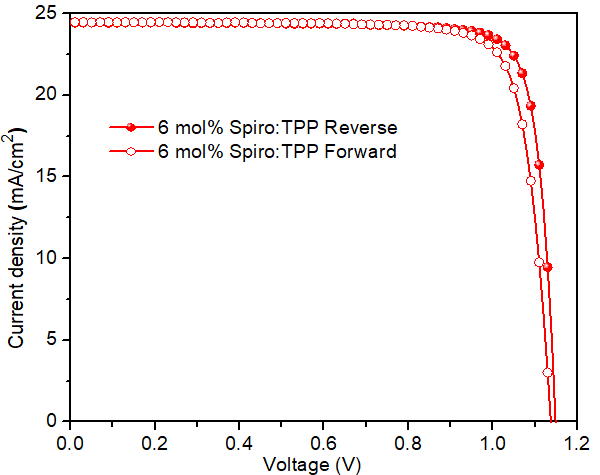

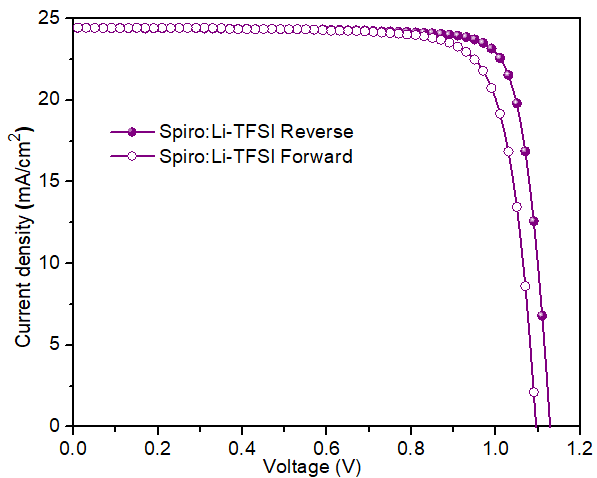


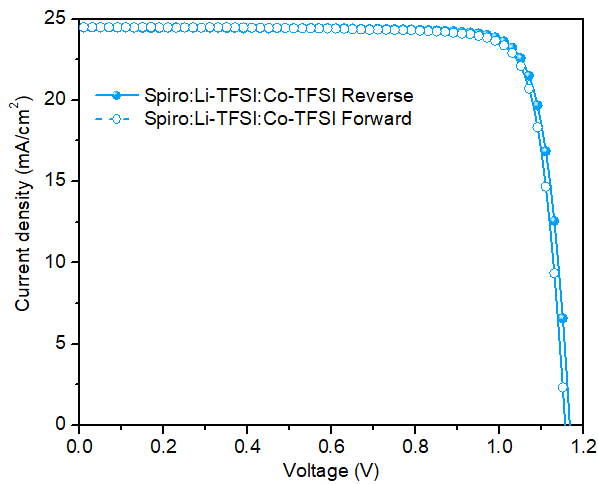


**Figure S8** The reverse and forward scans of *J*-*V* curves for devices based on Spiro:Li-TFSI, Spiro:TPP and Spiro:Li-TFSI:Co-TFSI.

**Figure S9** The stability of devices based on different dopants under 85 ℃ heating in the N_2_ filled glove box.

**Table S1.** Fitting parameters by *bi*-exponential decay function in TRPL of glass/perovskite substrates based on different HTMs.

| Films | A_1_ (%) | *τ*_1_ (ns) | A_2_ (%) | *τ*_2_ (ns) | *τ*_ave_ (ns)^a^ |
| --- | --- | --- | --- | --- | --- |
| FTO/perovskite/Spiro:Li-TFSI | 70.0 | 8.2 | 30.0 | 91.7 | 77.2 |
| FTO/perovskite/Spiro:TPP | 53.0 | 7.4 | 47.0 | 61.5 | 55.9 |
| FTO/perovskite/Li-TFSI | 76.1 | 58.3 | 23.9 | 358.3 | 255.9 |
| FTO/perovskite/TPP | 74.3 | 67.3 | 25.7 | 432.1 | 318.7 |

^a^Average decay time is calculated according to the equation: *τ*_ave_ = (A_1_*τ*_1_^2^+ A_2_*τ*_2_^2^)/(A_1_*τ*_1_+ A_2_*τ*_2_).

**Table S2.** Photocoltaic parameters of devices based on various doping TPP concentrations measured in different scan directions.

| Concentrations | Scan directions | *J*_sc_ (mA/cm^2^) | *V*_oc_  (V) | FF | PCE_max_  (%) | Average PCE  (%) |
| --- | --- | --- | --- | --- | --- | --- |
| Spiro-dopant free | reverse | 22.84 | 1.046 | 0.670 | 16.00 | 14. 8±1.2 |
|  | forward | 22.86 | 0.992 | 0.613 | 13.90 |  |
| TPP 2 mol% | reverse | 24.01 | 1.081 | 0.771 | 20.01 | 18.1±1. 9 |
|  | forward | 24.03 | 1.051 | 0.763 | 19.27 |  |
| TPP 4 mol% | reverse | 24.35 | 1.122 | 0.801 | 21.88 | 20.4±1.5 |
|  | forward | 24.37 | 1.103 | 0.782 | 21.02 |  |
| TPP 6 mol% | reverse | 24.46 | 1.149 | 0.820 | 23.03 | 21.8±1.3 |
|  | forward | 24.47 | 1.127 | 0.810 | 22.33 |  |
| TPP 8 mol% | reverse | 24.39 | 1.133 | 0.807 | 22.30 | 21.4±0.9 |
|  | forward | 24.43 | 1.114 | 0.794 | 21.61 |  |

**Table S3.** Photocoltaic parameters of champion devices based on Spiro:Li-TFSI and Spiro:TPP with different scan directions.

| Dopants | *J*_sc_ (mA/cm^2^) | *V*_oc_  (V) | FF | PCE  (%) |
| --- | --- | --- | --- | --- |
| Spiro:Li-TFSI reverse scan | 24.42 | 1.128 | 0.813 | 22.39 |
| Spiro:Li-TFSI forward scan | 24.45 | 1.096 | 0.798 | 21.38 |
| Spiro:TPP reverse scan | 24.46 | 1.149 | 0.820 | 23.03 |
| Spiro:TPP forward scan | 24.47 | 1.127 | 0.810 | 22.33 |
| Spiro:Li-TFSI:Co-TFSI reverse scan | 24.48 | 1.166 | 0.821 | 23.43 |
| Spiro:Li-TFSI:Co-TFSI forward scan | 24.50 | 1.154 | 0.814 | 23.01 |
